# Factors Influencing Self-Confidence and Willingness to Perform Cardiopulmonary Resuscitation among Working Adults—A Quasi-Experimental Study in a Training Environment

**DOI:** 10.3390/ijerph19148334

**Published:** 2022-07-07

**Authors:** Filip Jaskiewicz, Dawid Kowalewski, Ewa Kaniecka, Remigiusz Kozlowski, Michal Marczak, Dariusz Timler

**Affiliations:** 1 Emergency Medicine and Disaster Medicine Department, Medical University of Lodz, 90-419 Lodz, Poland; filip.jaskiewicz@umed.lodz.pl (F.J.); dawid.kowalewski@umed.lodz.pl (D.K.); ewa.kaniecka@stud.umed.lodz.pl (E.K.); remigiusz.kozlowski@umed.lodz.pl (R.K.); 2 Department of Management and Logistic in Healthcare, Medical University of Lodz, 90-419 Lodz, Poland; michal.marczak@umed.lodz.pl

**Keywords:** out-of-hospital cardiac arrest, cardiopulmonary resuscitation, CPR, resuscitation, education

## Abstract

**Background:** There is a potential relationship between the self-confidence and the willingness of bystanders to undertake resuscitation (CPR) and its training. The current guidelines increasingly focus on both the importance of the human factor and the fact that training programs should increase the willingness of bystanders to undertake resuscitation, which may have a direct impact on improving survival in out-of-hospital cardiac arrest (OHCA). Aim: The objective of the study was to analyze factors influencing the assessment of own skills crucial in basic life support (BLS) and the willingness to provide CPR to individual victims. **Methods:** A pre-test and post-test quasi-experimental design was used in this study. The data was collected from 4 December 2019 to 3 October 2020 in workplaces, during instructor-led BLS courses. Each intervention (training) consisted of a theoretical and a practical part. The program was focused both on the skills and the human factor. **Results:** Comparison of pre-test and post-test data concerning self-confidence scores of the ability to recognize OHCA among 967 participants demonstrated a significant difference (respectively, Me = 2.2, IQR [2–3] vs. Me = 3.4, IQR [3–4]; *p* = 0.000). Additionally, self-assessment scores for the ability to perform proper chest compressions between pre-test and post-test also differed significantly (respectively Me = 2.3, IQR [2–3] vs. Me = 3.3, IQR [3–4]; *p* = 0.000). A highly significant difference was found in the likelihood of changing the decision in favor of the willingness to undertake CPR for all types of victims, with the greatest difference found in relation to the willingness to conduct resuscitation on strangers (OR = 7.67, 95% CI 5.01–11.73; *p* < 0.01). **Conclusions:** Completing hands-on training has a highly significant, beneficial effect on the readiness to undertake resuscitation for all types of victims, strangers in particular. Training programs should place particular emphasis on developing readiness to undertake resuscitation for both those who have never been trained and those who had their last training more than one year ago.

## 1. Introduction

As early as the 1960s, Friedrich Wilhelm Ahnefeld initially described the Chain of Survival to increase the chances of survival in out-of-hospital cardiac arrest (OHCA) [1]. In the decades that followed, this concept was modified and updated [2,3,4,5]. Early recognition of sudden cardiac arrest (SCA) and activation of the emergency response system (EMS) is the first link in the Chain of Survival. Immediate and high-quality bystander cardiopulmonary resuscitation (CPR) after effective identification of SCA, which is the second link, can increase the chance of survival from OHCA. It is currently known that such measures can be associated with up to a threefold increase in survival and, extremely importantly, favorable neurological outcomes [6,7,8,9,10].

As originally conceived, the chain is no stronger than its weakest link. From this perspective, the role of a bystander is crucial in rescuing SCA victims. The 2021 guidelines of the European Resuscitation Council strongly emphasize that the main goals of resuscitation training should include increased frequency of resuscitation in OHCA cases. Of course, they also constantly promote effective basic life support and automated defibrillation (BLS AED), and the fastest possible activation of EMS [11]. The objectives of these measures are clear, however, there are still research gaps about the most effective ways to teach technical elements of resuscitation. Questions about methods, didactic tools, frequency of refresher training sessions or their impact on the actual skills and effectiveness self-assessment among participants need to be answered [11].

The current guidelines also increasingly focus on both the importance of the human factor and the fact that training programs should increase the readiness and willingness of bystanders to undertake resuscitation. It may have a direct impact on improving survival in OHCA [11,12]. Although the effectiveness of the Chain of Survival itself is unquestionable, it should be remembered that the entire process must be initiated by a person. Accepting this fact has contributed to increasing research on the identification of barriers preventing bystanders from undertaking CPR [13,14,15,16,17,18]. Many of them are non-modifiable or hardly modifiable factors, such as age, gender, low social status, individual factors (physical weakness and disability), the inability to change the patient’s position or strong emotional factors (panic) [19].

A literature review also indicates a potential relationship between the self-confidence and the willingness of bystanders to undertake resuscitation and CPR training [20,21,22,23,24,25,26]. However, both the methodology and the characteristics of study groups are very diverse. Although SCA at workplace is relatively rare, it is characterized by both higher survival and more favorable neurological outcomes [27,28,29,30,31,32]. A potential full recovery and resumption of professional activity are also important. For this reason, our attention was drawn to the modifiable training factors influencing the self-confidence in key skills and the readiness to undertake CPR by first aiders at a workplace.

## 2. Aims

The general aim of this study was to analyze factors influencing the assessment of own skills crucial in basic life support (BLS) and the willingness to provide CPR to individual victims.

Specific aims were also set to evaluate:

1. The influence on the ability to recognize the symptoms of SCA and perform correct chest compressions was analyzed in the context of the following factors:
  - Previous participation in a first aid training in general;
  - The time that has elapsed since previous first aid training;
  - Previous hands-on-training of checking for consciousness and the presence of normal breathing in the past (CBA skill: consciousness and breathing assessment);
  - Previous hands-on-training of chest compressions on a manikin (CCs skill: chest compressions);
  - Completing the course in accordance with the planned methodology.
2. Whether the willingness of participants to undertake resuscitation on individual victims depends on completing the course in accordance with the planned methodology;
3. How the current training changes participants’ decision to undertake CPR on individual victims if previous training history is taken under consideration.

## 3. Materials and Methods

### 3.1. Study Design and Setting

**Figure 1:**
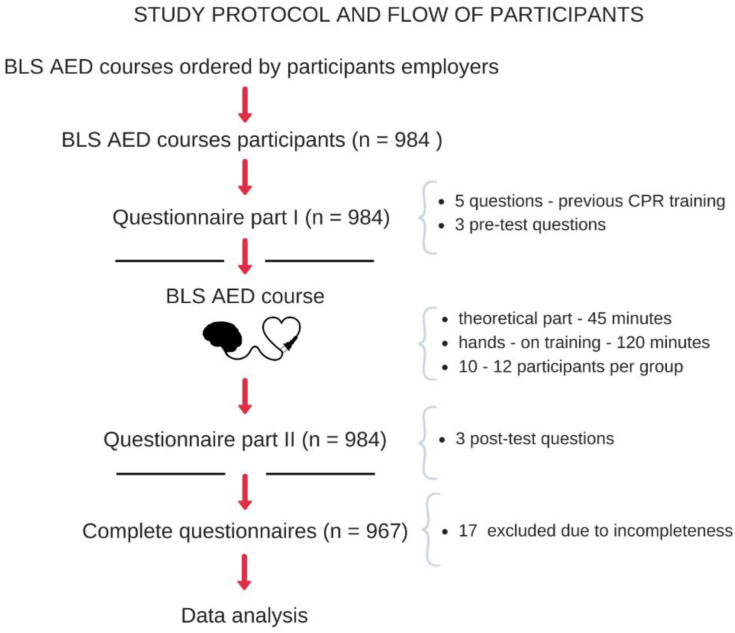
Study protocol and the flow of participants. BLS AED-effective basic life support and automated defibrillation; CPR-cardiopulmonary resuscitation.

A pre-test and post-test quasi-experimental design was used in this study. The protocol and the flow of participants are presented in Figure 1. The data was collected from 4 December 2019 to 3 October 2020 in workplaces, during commercial BLS AED courses ordered by participants employers. The training with the adopted methodology was an intervention in the study. The program was based on the 2015 European Resuscitation Council (ERC) Guidelines focused both on the skills and the human factor. Each training consisted of a theoretical part in the form of a multimedia presentation (45 min) and a practical part (120 min). The lecture included a simulation video of a man collapsing due to cardiac arrest and presenting with gasping (20 s), and a detailed discussion of cardiac arrest symptoms. The practical part consisted of a demonstration and exercises to practice the assessment of consciousness and breathing (on other trainee and a manikin). Then, a demonstration (Brayden Adult Advanced manikin; Innosonian Inc., Seoul, Korea) and BLS AED training (Resusci Anne QCPR manikin with real time feedback; Laerdal, Stavanger, Norway) were performed. There were no more than 10–12 participants in each training group. The study received a positive opinion of the Bioethics Committee at the Medical University of Lodz (number RNN/222/19/KE).

### 3.2. Participants

The study was conducted among employees appointed by their employers to provide first aid in workplaces (first aiders). While participation in course was ordered by employer, participation in the study was voluntary. Inclusion criteria were as follows: full attendance in the BLS AED course and a complete set of information in the questionnaire.

### 3.3. Data Sources/Measurement

A pre-test and post-test survey questionnaire (Supplementary Materials) was used as the research tool to collect the data. The process of its creation and evaluation was as follows:

1. Stage I.—Literature review to identify questions used in research on similar topics, including: literature reviews—total n = 6, (1 was rejected due to the lack of direct reference to the topic of the work); original articles, in total n = 51 (13 were rejected due to lack of direct reference to the topic of the work or to a very specific population); guidelines and international recommendations = 4—The International Liaison Committee On Resuscitation Education Implementation And Teams Task Force, American Heart Association and European Resuscitation Council Guidelines.
2. Stage II.—Critical analysis of questions used in other researchers’ questionnaires and their verification in terms of consistency with the goals of the presented study.
3. Stage III.—Creating a questionnaire appropriate to verify the chosen aims of the study.
4. Stage IV.—Critical content evaluation by an experienced subject matter experts (n = 3)—removal of one question that was considered divergent from the chosen study aims; adding a question regarding the general characteristics of the group concerning the voivodeship of residence of the respondents.
5. Stage V.—Presentation of the questionnaire in a pilot form to 30 respondents (debriefing respondents to obtain additional information about their views of the questions/concepts) participating in commercial resuscitation courses to evaluate the questionnaire in terms of whether:
  - Closed questions provide at least one answer choice that would apply to every respondent;
  - Questions were interpreted in the same manner by all the respondents;
  - Answer choices to be selected correct;
  - Questionnaire creates a positive impression, thus motivating people to respond to the question;
  - Finally, whether any aspect of the questionnaire suggests any bias from the researcher.

As a result of this evaluation, minor changes were made to the wording of the question relating to the type of casualties for whom the subjects would undertake CPR: the words “colleague/neighbor” were replaced with the words “person you know”; the option to choose “I would not undertake CPR”, replaced with “I would not undertake CPR, regardless of the circumstances and who the victim is”.

Stage V. completed the evaluation process of the questionnaire, and due to its simplicity and short form, the more detailed validation of the research tool was abandoned or not relevant to the evaluated questionnaire (e.g., Cronbach’s alpha test).

Finally, the participants were asked to complete the first part of the questionnaire containing:

Two questions on age and place of residence, 5 questions on the details of previous CPR training, and 3 pre-test questions before the training. After the training, the participants again answered the same 3 questions in the post-test: self-confidence in recognition cardiac arrest symptoms; self-confidence in providing proper chest compressions (in both cases a scale 1–4 was used: 1—not able, 2—not sure, 3—able, 4—definitely able) and a multiple-choice question on the willingness to undertake CPR on different victims (family member, child, person you know, stranger).

### 3.4. Statistical Analysis

Statistical analysis of the data was performed using Statistica v. 13. Elements of descriptive statistics were used to determine the mean, median, minimum, maximum and standard deviation. The Shapiro–Wilk normality test was used to check the distribution. Specific aims were verified using non-parametric tests. The Wilcoxon’s pair order test was used for the analysis of 2 ordinal and quantitative variables with a distribution inconsistent with the normal distribution. The Friedman test and the Friedman post hoc test were used for more than two groups. Pre- and post-test declarations on the willingness of participants to undertake resuscitation on individual victims were compared with the McNemar test and changes depending on the group were compared with the Chi-squared dependence test. Quantitative variables are presented using basic descriptive statistics: the arithmetic mean (x), standard deviation (SD), median (Me), interquartile range [IQR] and percentages (%). Test probability at *p* < 0.05 was considered significant and test probability at *p* < 0.01 was considered highly significant.

## 4. Results

Data from 967 out of 984 voluntary questionnaires (17 were excluded due to incompleteness) obtained from working adults were used for the analysis. The participants came from 14 out of 16 voivodeships in Poland. The mean age of the respondents was 36 ± 8.7 years (Me = 36; Min = 19; Max = 64). A total of 733 (76%) participants attended a first aid training course in the past. Of these, only 620 (85%) trained the skills of consciousness and normal breathing assessment (on a manikin or other trainee), 630 (86%) trained chest compressions on an adult manikin and 360 (49%) on a child’s manikin. Only 346 (47%) participants trained the use of an automatic external defibrillator during previous training.

The analysis of the pre-test data showed a highly statistically significant relationship between the self-assessment of the ability to recognize cardiac arrest symptoms depending on whether a given participant had received first aid training in the past (*p* = 0.000). Highly statistically significant differences were also found in the time elapsed from the training among the respondents who participated in such training (Table 1). The only two groups in the comparison of which no statistically significant difference was found were: Yes, in 1–2 years/Yes, in >2 years (*p* > 0.05).

**Figure 2:**
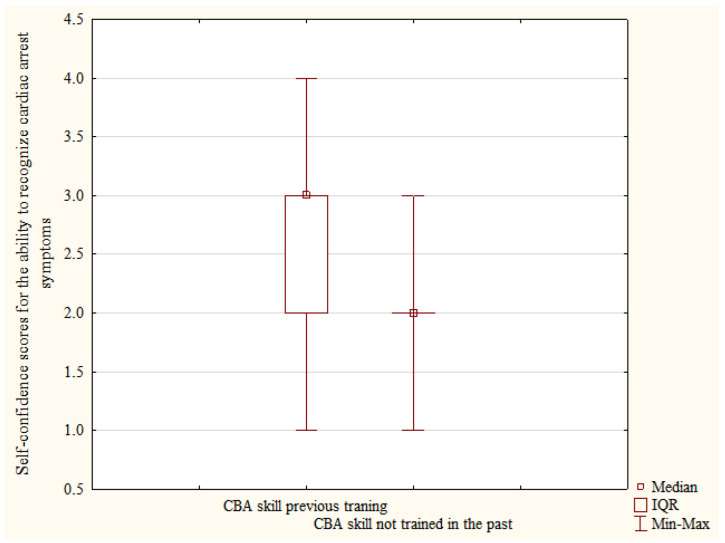
Self-confidence scores-recognition of cardiac arrest symptoms between study participants who previously trained their skills of assessing consciousness and the presence of normal breathing (CBA skill) and those who did not pre-test data.

Among those participants who had completed a first aid course in the past, a statistically significant difference was also found in the results of self-confidence scores for the ability to recognize cardiac arrest symptoms between participants who had hands-on-training on assessing consciousness and the presence of normal breathing (CBA skill) on a manikin or a course participant (n = 620) and those who did not (n = 113), respectively, Me = 3.0, IQR [2–3] vs. Me = 2.0, IQR [2–2]; *p* < 0.001-Figure 2.

Pre-test data analysis showed a highly significant relationship between the self-confidence scores for the ability to perform proper chest compressions depending on whether a given participant had received first aid training in the past (*p* = 0.000). Highly statistically significant differences were also found in the time that elapsed from the training among the respondents who participated in such training (Table 2). The only two groups in comparison of which no statistically significant difference was found were again: Yes, in 1–2 years/Yes, in >2 years (*p* > 0.05).

**Figure 3:**
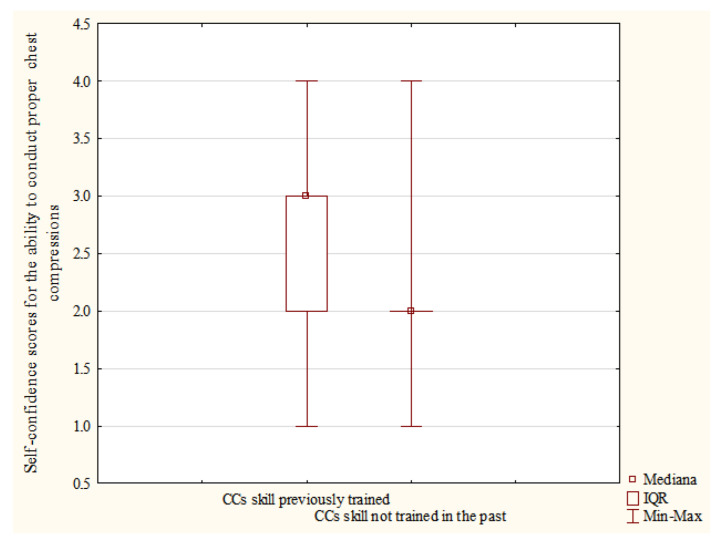
Self-confidence scores-performing proper chest compressions between respondents who previously practiced chest compressions (skill CCs) on a manikin and those who did not pre-test data.

Among those participants who had completed first aid course in the past, a statistically significant difference was also demonstrated in the results of self-confidence scores for the ability to perform proper chest compressions (CCs skill) between respondents who previously trained chest compressions on a manikin (n = 630) and those who did not (n = 103), respectively, Me = 3.0, IQR [2–3] vs. Me = 2.0, IQR [2–2]; *p* < 0.001-Figure 3.

Comparison of pre-test and post-test data (before and after the current training) concerning self-confidence scores of the ability to recognize cardiac arrest symptoms demonstrated a highly statistically significant difference (respectively, Me = 2.2, IQR [2–3] vs. Me = 3.4, IQR [3–4]; *p* = 0.000). An analysis of self-assessment scores for the ability to perform proper chest compressions between pre-test and post-test also differed significantly (respectively, Me = 2.3, IQR [2–3] vs. Me = 3.3, IQR [3–4]; *p* = 0.000).

The last question in both pre-test and post-test was aimed to determine the number of study group participants who declared that they would undertake CPR on individual victims. In all cases, statistically significant differences were found in participants’ declarations before and after the current BLS AED training (Table 3).

In order to assess the impact of the current training on the frequency of changing the decision from NO—I will not start CPR to YES—I will start CPR (compared to YES—I will start CPR to NO—I will not start CPR), an analysis was conducted for individual types of victims (Table 4). In relation to the entire study group, a highly statistically significant difference was found in the probability of changing the decision in favor of the willingness to undertake CPR for all types of victims.

Regarding past training, the positive effect of the current training in changing decisions and willingness to undertake CPR was most evident with participants trained more than 2 years ago (Table 4). Those who had never been trained in the past significantly more often changed their decision in favor of starting CPR in children, strangers and people they know (in this case, a highly significant change of mind is observed (*p* < 0.01), but the odds ratio cannot be calculated because there have been no cases of change of opinion from Yes to No). Study participants who had been trained in the period 1–2 years changed their minds in favor of CPR only in the case of a stranger. Among those who received training in the last year, no significant impact of the current training on changing their decision to start CPR in any type of victim was observed.

## 5. Discussion

Sudden cardiac arrest (SCA) is a serious public health problem associated with low survival rates. This mainly applies to communities with a low bystander CPR rates [4,6]. Increasing bystanders’ readiness to respond to cardiac arrest is of critical importance for improving survival [6,7,8,9,10,11]. Resuscitation training programs that focus not only on improving practical skills but also increasing the readiness and willingness of bystanders to undertake resuscitation may directly contribute to an increased survival in OHCA [10,11]. This issue is of increasing interest among researchers. Many studies have confirmed the relationship between training and readiness to undertake CPR and the self-evaluation of skills among bystanders [20,21,22,23,24,25,26]. The percentage of individuals who have ever attended a CPR training course varies greatly depending on the country: 34.8–37.6% in China [23,33], 45.7% in Taiwan [22], 53% in Crimea [24], 76% in Sweden [34], 89% in Germany [35], and 90% in Norway [36]. This value was 76% in the presented study. Unfortunately, these results are difficult to compare, mainly due to significant differences in the methodology and characteristics of study groups.

According to the “Intention-Focused” paradigm by Panchal et al., whose purpose is to improve bystander CPR performance, self-reported skills have a significant impact on the willingness to provide first aid, and therefore resuscitation undertaken by a bystander [37]. This study analyzed the impact of selected factors related to the participants’ self-evaluation in terms of two elementary activities of the BLS algorithm in accordance with the chosen methodology [4,5,11]. Consistent results were obtained for both the self-reported ability to recognize SCA and correctly perform chest compressions. Previous participation in training (compared to those who had never been trained) had a highly significant (*p* < 0.001) positive impact on self-evaluation of both analyzed skills. In the group of previously trained respondents, the highest self-rating was observed among participants who completed first aid courses in the last year. Greater elapse of time from the training session was not associated with any significant differences in self-evaluation (groups: Yes, 1–2 years vs. Yes, >2 years, *p*> 0.05). These findings are consistent with the ILCOR Consensus of Science and Treatment Recommendation [12]. They indicate that both the BLS skills and confidence and readiness to perform CPR decline 3–12 months after initial CPR training. It is worth noting that these recommendations are still weak, based on low reliability evidence. This is due to, among other things, low-quality research and contradictory scientific reports on the impact of previous training on self-reported skills or self-confidence, obtained by various authors [24,26,38,39].

The definition, teaching methods used, subject matter and training duration vary depending on the study both in relation to the concepts of first aid training and basic life support [19,20,21,22,23,24,25,26,40]. For the purposes of the presented analysis, not only the previous training itself was assessed, but it was also verified whether it involved hands-on training in assessing consciousness and normal breathing (the CBA skill: assessment of consciousness and breathing) and hands-on training of chest compressions with training manikin (CCs skill: chest compressions). In both cases, these factors were highly significant and had positive impact on the self-evaluation of particular skills among the respondents. This means that hands-on training has the greatest impact on the increased self-rating of skills, and thus self-confidence.

The study also found a statistically significant increase in the self-rating of own skills in the recognition of cardiac arrest throughout the study group after the current training (pre-test: Me = 2.2, IQR [2–3] vs. post-test: Me = 3.4, IQR [3–4], respectively; *p* = 0.000) and performing correct chest compressions (pre-test: Me = 2.3, IQR [2–3] vs. post-test: Me = 3.3, IQR [3–4]; *p* = 0.000).

The obtained results are consistent with the conclusions of González-Salvado et al., who performed a systematic review of the literature on training adult laypeople in basic life support [40]. The authors of the review emphasized that instructor-led hands-on training supported by feedback devices is superior to other methods. It is worth noting, however, that the mere fact of past training already contributes to the self-evaluation or the willingness to use skills, with overall positive results regardless of the teaching method used, in many of the studies analyzed in the review [40]. Therefore, it seems reasonable to teach CPR through hands-on training. If not feasible, other forms of teaching can also produce benefits in terms of self-evaluation of skills and self-confidence [11,12].

Considering the latest ILCOR guidelines [12] and the “Intention-Focused” paradigm by Panchal et al. [37], the present study also analyzed the impact of training on the willingness to undertake resuscitation. Literature analysis shows that it may differ depending on the victim involved [19,22,23,24,25,33,38,39]. For this reason, the willingness to undertake CPR was assessed separately for a family member, a child, a known person, and a stranger.

For all types of victims, the willingness to undertake resuscitation was high and comparable with the results obtained by Birkun et al. or Chen et al. [24,41]. The analysis of the pre- and post-test results throughout the study group indicates a highly significant (*p* < 0.01), beneficial effect of the currently completed training on increasing the percentage of respondents willing to perform resuscitation for all types of victims. The obtained results are consistent with the reports of other authors who assessed the impact of training on the willingness to undertake resuscitation [26,33,39].

As in the case of other studies, the largest number of respondents (pre-test: 94.2% vs. post-test: 96.5%) expressed readiness to perform CPR in a family member [22,24,33,39,42]. Worth noticing, that the highest percentage increase in the willingness to undertake CPR after training was found for a stranger (pre-test: 74.8% vs. post-test: 91.3%). This study did not analyze the barriers to undertaking CPR, but those indicated most often by other authors may be of particular importance in the case of an unknown victim (stranger): causing harm, fear of legal problems or contracting an infectious disease [19,22,24].

The present study also analyzed the impact of the current training on the frequency of changing the decision from NO—I will not start CPR to YES—I will start CPR (compared to YES—I will start CPR to NO—I will not start CPR) for individual types of victims. Throughout the study group, a highly statistically significant difference was found in the likelihood of changing the decision in favor of the willingness to undertake CPR for all types of victims, with the greatest difference again found in relation to the willingness to conduct resuscitation on strangers (OR = 7.67, 95% CI 5.01–11.73; *p* < 0.01). Many studies indicate that this group receive bystander CPR less frequent. The reasons for this fact are seen in the greatest number of barriers among witnesses. Therefore, an intervention in the form of training has the most visible effect on eliminating barriers in this group of patients [19,22,23,24,25,33,38,39,40].

In the analysis considering past training history, the positive impact of current training on changing the decision to undertake CPR was most evident for participants trained >2 years ago. They were more likely to switch from NO to YES than YES to NO for all types of victims. Those who had never been trained in the past were significantly more likely to change their decision to perform CPR in children, strangers, and people they know. Study participants trained 1–2 years ago only changed their minds in favor of CPR for a stranger. Among those who underwent training in <1 year, the current training did not have a significant impact on changing the decision about the willingness to undertake CPR in any type of victim. Again, these findings are consistent with the recent ILCOR Recommendations [11,12], which indicate on negative correlation between readiness to perform CPR and time passing after initial CPR training (over 3–12 months). Additionally, González-Salvado et al. and Scapigliati et al., in their literature reviews, emphasize the role of repeating training and community initiatives [10,40]. Most educational interventions in the studies analyzed by Scapigliati et al. had impact on the frequency of willingness and actual performance of bystander CPR. Worth noticing, this benefit was more frequent when the type of initiative was a ‘bundle’ of interventions compared to single training or mass-media initiatives.

The obtained results allow for the conclusion that the currently completed training had the greatest impact on the willingness to undertake resuscitation among the respondents who had not been trained in the past and those with >1 year elapsed since the previous training course. As for the type of victim, the greatest benefit from the impact of the current training on the willingness to undertake resuscitation was observed for strangers. In that case not only the greatest increase in the willingness to undertake resuscitation after training was shown in the entire study group, but also the highest frequency of changing decisions after training in all participants untrained in the past and those who had training in the period of >1 year.

As for the specific aims, the results of the present study indicate a significant impact of past training and hands-on training on both the self-reported key BLS skills and the readiness to undertake resuscitation in all types of victims. However, both self-confidence and readiness decline over time after training. Scientific reports on this issue are very diverse, mainly due to the selection of study groups and methodology. Furthermore, an ideal model for CPR training and refresher training process aimed at maintaining skills or knowledge is still missing [11]. It is undeniable, however, that the impact of the human factor and the target of training programs on improving the readiness to undertake resuscitation by bystanders may have a direct impact on increasing survival in OHCA [10,11,12]. For this reason, further research assessing the impact of training on self-confidence and readiness to undertake resuscitation should seek to develop guidelines not only for the frequency and method of training, but also effective teaching techniques and tools that will allow for improving both self-confidence and willingness to help in sudden cardiac arrest.

### Limitations

This study had several limitations. One of the main ones concerned the design of the study and the inability to plan and calculate the study sample. Therefore, it was assumed that in order to achieve statistical significance, the study will include successively reported by employers groups of employees until the number of respondents meeting the inclusion criteria reaches 1000. As the data were collected among participants of commercial BLS trainings, AED was difficult to predict and determine the study duration. Unfortunately, due to the development of the SARS-CoV-2 pandemic and the limitations related to the organization of group meetings, it was not possible to reach the full 1000 number of respondents. At the same time, the outbreak of the pandemic itself could have a potential impact, both favorable or unfavorable, on the obtained results. Additionally, the impact of the information about the participation in first aid training in the past may be different for each participant as the form of this training, its duration and the detailed program were not taken into consideration.

## 6. Conclusions

The self-confidence in recognition of cardiac arrest and performance of proper chest compressions is positively influenced by previous training, the time elapsed since the last training (no more than 1 year), and previous practical training of these skills. Additionally, completion of hands-on training significantly contributes to improved self-assessment of these key skills. Completing hands-on training has a highly significant, beneficial effect on the readiness to undertake resuscitation for all types of victims, strangers in particular. Training programs should place particular emphasis on developing readiness to undertake resuscitation for both those who have never been trained and those who had their last training more than one year ago.

## Figures and Tables

**Table 1:** Self-confidence scores for the ability to recognize cardiac arrest symptoms depending on previous training.

Participation in First Aid Course in the Past	Yes, <1 Year	Yes, 1–2 Years	Yes, >2 years	No Never
	n = 220	n = 105	n = 408	n = 235
**PRE-TEST Recognizing the symptoms of cardiac arrest?** **(Scale: 1-not able, 2-not sure, 3-able, 4-definitely able)**	Me, [IQR]			
	2.8, [3–3]	2.3 [2–3]	2.3, [2–3]	1,8, [1–2]
***p*-value**	0.000			

**Table 2:** Self-confidence scores for the ability to perform proper chest compressions depending on previous training.

Participation in the First Aid Course in the Past	Yes, <1 year	Yes, 1–2 years	Yes, >2 years	No, Never
	n = 220	n = 105	n = 408	n = 235
**PRE-TEST Conducting proper chest compressions?** **(Scale: 1-not able, 2-not sure, 3-able, 4-definitely able)**	Me, [IQR]			
	3.1, [3–3]	2.4, [2–2]	2.3, [2–3]	1.6, [1–2]
***p* value**	0.000			

**Table 3:** Comparison of the pre-test and post-test results on the number of participants (n = 967) who declared that they would undertake CPR on individual victims (family member, child, person you know, stranger) multiple choice question.

Victim Characteristic	Pre-Test, (%)	Post-Test, n (%)	*p* Value
Family member	911 (94.2)	933 (96.5)	0.015
Child	833 (86.1)	903 (93.4)	0.000
Person you know	887 (91.7)	927 (95.9)	<0.001
Stranger	723 (74.8)	883 (91.3)	0.000

**Table 4:** The probability of changing the decision to undertake CPR in individual victims after current training.

Victim Characteristic and Previous Training History	OR	−95%CI	+95 CI	*p* Value
Family member				
Regardless of training history	2.1	1.23	3.58	0.007
No never	4	0.85	18.84	0.113
Yes, <1 year	0.8	0.32	2.03	0.813
Yes, 1–2 years	3	0.61	14.86	0.289
Yes, >2 years	3.33	1.34	8.3	0.010
Child				
Regardless of training history	4.5	2.77	7.31	<0.001
No never	7.5	2.64	21.29	<0.001
Yes, <1 year	1.6	0.73	3.53	0.326
Yes, 1–2 years	4	0.85	18.84	0.113
Yes, >2 years	9	3.2	25.29	<0.001
Person you know				
Regardless of training history	3.22	1.9	5.47	<0.001
No never	NA	NA	NA	<0.001
Yes, <1 year	1	0.45	2.23	0.838
Yes, 1–2 years	4	0.85	18.84	0.113
Yes, >2 years	6	2.08	17.29	<0.001
Stranger				
Regardless of training history	7.67	5.01	11.73	<0.001
No never	27	6.58	110.74	<0.001
Yes, <1 year	1.63	0.87	3.03	0.164
Yes, 1–2 years	7	1.59	30.08	0.006
Yes, >2 years	22.5	8.26	61.25	<0.001

## Data Availability

The data presented in this study are available on request from the corresponding author.

## Supplementary Materials

The following supporting information can be downloaded at: https://www.mdpi.com/article/10.3390/ijerph19148334/s1. Pre-test and post-test survey questionnaire.
