# Supplementary material for: Factors Influencing Self-Confidence and Willingness to Perform Cardiopulmonary Resuscitation among Working Adults—A Quasi-Experimental Study in a Training Environment
Source: Int J Environ Res Public Health. 2022 Jul 7;19(14):8334. doi: 10.3390/ijerph19148334 (PMC9322983; doi:10.3390/ijerph19148334)
Supplement: Supplementary file 1 [file ijerph-19-08334-s001.zip › ijerph-1779880-supplementary.pdf]

Survey form

Hello. I am asking you to take part in a study conducted at the [redacted] The study is directed only to working adults, not related to medicine. On its basis, a scientific publication will be created on "Factors influencing self-confidence and willingness to perform CPR in working adults". The survey form consists of 13 questions, it is anonymous and it will take you about 2 minutes to complete it. Participation in the study is voluntary and anonymous. The survey consists of two parts. Complete Part I (page 1.) before the training and Part II (page 2.) after the training. Thank you for your time.

PART I
Please complete this part now.

1. **Age**

……………………….

2. **Voivodeship**

………………………………..

3. **Have you participated in a first aid course in the past?**

- ☐ **NO, never (in this case go to question 8)**
- ☐ **YES, within 1 year**
- ☐ **YES, in 1-2 years**
- ☐ **YES, in > 2 years**

4. **Have you trained the skills of consciousness and normal breathing assessment (on a manikin or other trainee)**

- ☐ **YES**
- ☐ **NO**

5. **Have you trained chest compressions on an adult version manikin?**

- ☐ **YES**
- ☐ **NO**

6. **Have you trained chest compressions on an child version manikin?**

- ☐ **YES**
- ☐ **NO**

7. **Have you trained the use of an automatic external defibrillator?**

- ☐ **YES**
- ☐ **NO**

8. **For which of the following victims would you undertake a consciousness assessment, a breath assessment, and a provide resuscitation? (select all that apply):**

- ☐ Family member
- ☐ Child
- ☐ Person you know
- ☐ Stranger
- ☐ I would not undertake CPR, regardless of the circumstances and who the victim

9. **Do you think, you can recognize the symptoms of cardiac arrest? How do you rate the effectiveness of your skills in this matter?**

1------------------------------2----------------------------3------------------------------4
not able not sure able definitely able

10. **Do you think you can perform proper chest compressions? ? How do you rate the effectiveness of your skills in this matter?**

1------------------------------2----------------------------3------------------------------4
not able not sure able definitely able

PART II
Please complete this part only after completing the training.

11. For which of the following victims would you undertake a consciousness assessment, a breath assessment, and a provide resuscitation? (select all that apply):

- ☐ Family member
- ☐ Child
- ☐ Person you know
- ☐ Stranger
- ☐ I would not undertake CPR, regardless of the circumstances and who the victim is

12. Do you think, you can recognize the symptoms of cardiac arrest? How do you rate the effectiveness of your skills in this matter?

1-----------------------------2----------------------------3-----------------------------4
not able not sure able definitely able

13. Do you think you can perform proper chest compressions? ? How do you rate the effectiveness of your skills in this matter?

1-----------------------------2----------------------------3-----------------------------4

not able not sure able definitely able
